# Gyrase and Topoisomerase IV: Recycling Old Targets for New Antibacterials to Combat Fluoroquinolone Resistance

**DOI:** 10.1021/acsinfecdis.4c00128

**Published:** 2024-04-02

**Authors:** Jessica A. Collins, Neil Osheroff

**Affiliations:** † Department of Biochemistry, Vanderbilt University School of Medicine, Nashville, Tennessee 37232, United States; ‡ Department of Medicine (Hematology/Oncology), Vanderbilt University School of Medicine, Nashville, Tennessee 37232, United States

**Keywords:** gyrase, topoisomerase IV, fluoroquinolone resistance, novel bacterial topoisomerase inhibitors, triazaacenaphthylenes, spiropyrimidinetriones

## Abstract

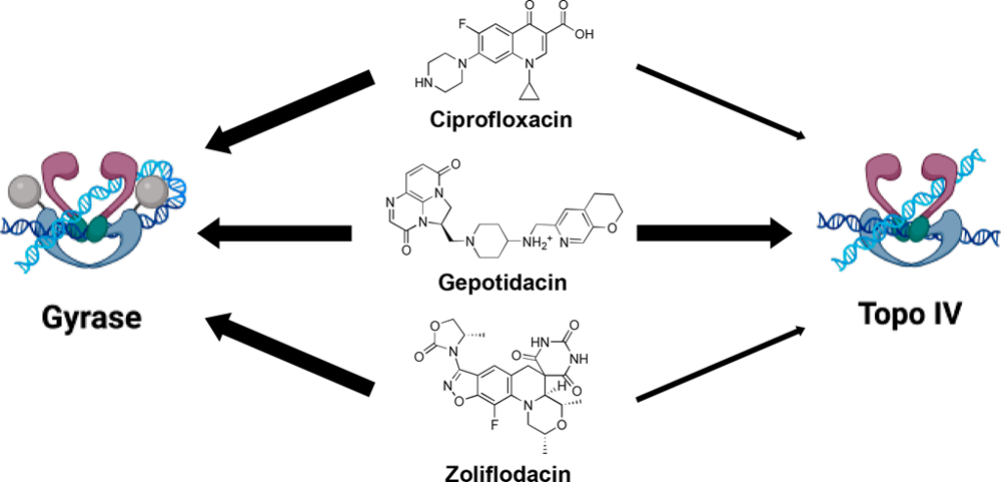

Beyond their requisite functions in many critical DNA processes, the bacterial type II topoisomerases, gyrase and topoisomerase IV, are the targets of fluoroquinolone antibacterials. These drugs act by stabilizing gyrase/topoisomerase IV-generated DNA strand breaks and by robbing the cell of the catalytic activities of these essential enzymes. Since their clinical approval in the mid-1980s, fluoroquinolones have been used to treat a broad spectrum of infectious diseases and are listed among the five “highest priority” critically important antimicrobial classes by the World Health Organization. Unfortunately, the widespread use of fluoroquinolones has been accompanied by a rise in target-mediated resistance caused by specific mutations in gyrase and topoisomerase IV, which has curtailed the medical efficacy of this drug class. As a result, efforts are underway to identify novel antibacterials that target the bacterial type II topoisomerases. Several new classes of gyrase/topoisomerase IV-targeted antibacterials have emerged, including novel bacterial topoisomerase inhibitors, *Mycobacterium tuberculosis* gyrase inhibitors, triazaacenaphthylenes, spiropyrimidinetriones, and thiophenes. Phase III clinical trials that utilized two members of these classes, gepotidacin (triazaacenaphthylene) and zoliflodacin (spiropyrimidinetrione), have been completed with positive outcomes, underscoring the potential of these compounds to become the first new classes of antibacterials introduced into the clinic in decades. Because gyrase and topoisomerase IV are validated targets for established and emerging antibacterials, this review will describe the catalytic mechanism and cellular activities of the bacterial type II topoisomerases, their interactions with fluoroquinolones, the mechanism of target-mediated fluoroquinolone resistance, and the actions of novel antibacterials against wild-type and fluoroquinolone-resistant gyrase and topoisomerase IV.

The bacterial type II topoisomerases, gyrase and topoisomerase IV, are the targets for the fluoroquinolone class of antibacterials.^1−7^ Members of this class include drugs such as ciprofloxacin, moxifloxacin, and levofloxacin.^5^ Fluoroquinolones are one of the most successful classes of antibacterials introduced into the clinic due to their high oral bioavailability, good tissue distribution, and broad-spectrum activity against Gram-negative, Gram-positive, and atypical (with regard to Gram staining) pathogens.^5,8−11^ This class of antibacterials has been listed by the World Health Organization (WHO) as one of their five “highest priority” critically important antimicrobials for treatments of infections in humans.^10^

Unfortunately, the clinical use of fluoroquinolones has been undermined by target-mediated resistance caused by specific mutations in gyrase and topoisomerase IV.^1,3,7,9^ Therefore, to take advantage of these validated targets, efforts have been made to identify new drug classes that interact with the type II enzymes but utilize different amino acid residues for their binding.^6,12−19^ As a result, recent drug discovery programs have led to the development of novel antibacterial classes that target gyrase and topoisomerase IV.^13,14,16−18^ Phase III clinical trials that utilized two members from these classes, gepotidacin and zoliflodacin, have been concluded with positive outcomes.^20−24^

Because of the reemergence of the bacterial type II topoisomerases as targets for new classes of advanced clinical candidates,^9,25,26^ it is important to understand how these antibacterials affect the activities of gyrase and topoisomerase IV. Therefore, this review will describe the catalytic mechanism and cellular activities of the bacterial type II topoisomerases, their interactions with fluoroquinolones, the mechanism of target-mediated fluoroquinolone resistance, and the actions of novel classes of gyrase/topoisomerase IV-targeted antibacterials.

## Bacterial Type II Topoisomerases

Gyrase was discovered in 1976 and was the first type II topoisomerase to be described.^27^ The enzyme was identified by its ability to convert relaxed closed-circular DNA substrates to negatively supercoiled (i.e., underwound) molecules.^27^ Gyrase is found in all bacterial species and is essential for life.^28^ For 14 years, gyrase was believed to be the only type II topoisomerase in bacterial cells. However, in 1990, the genes encoding a second type II topoisomerase, topoisomerase IV, were identified from a genetic screen for mutations that impeded chromosome partitioning in *Escherichia coli*.^29^ On the basis of sequencing and enzymology studies, topoisomerase IV was determined to be homologous to gyrase and utilize a similar reaction mechanism (detailed below).^28−30^

Most bacterial species encode topoisomerase IV in addition to gyrase, and in those species, both enzymes are essential.^28^ In contrast, some species such as *Mycobacterium tuberculosis, Helicobacter pylori*, and *Treponema pallidum* (the causative agents of tuberculosis, stomach ulcers, and syphilis, respectively) encode only gyrase.^31−35^ It has been postulated that in these species, gyrase has also taken over the critical biological functions of topoisomerase IV.^34,35^

**Figure 1:**
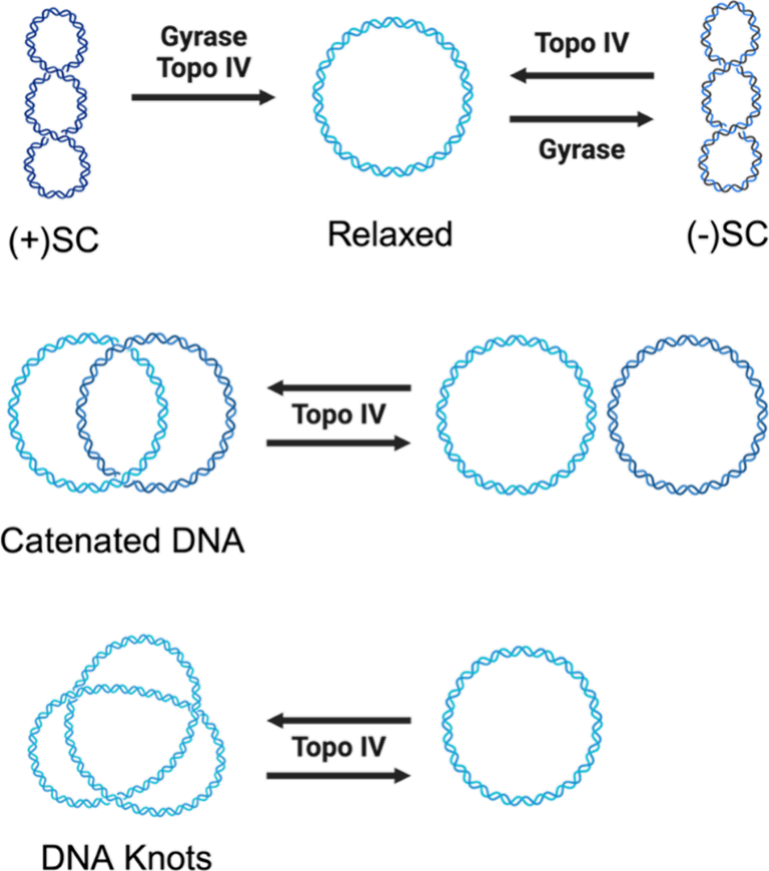
DNA strand passage activities of gyrase and topoisomerase IV. Gyrase primarily modulates the superhelical state of the bacterial genome by relaxing (i.e., removing) positive supercoils and generating negative supercoils in relaxed DNA. Topoisomerase IV primarily removes tangles (catenanes) and knots from the genetic material but can also relax positive and negative DNA supercoils.

As discussed below, gyrase and topoisomerase IV utilize a double-stranded DNA passage reaction to control cellular levels of DNA over- and under-winding (i.e., positive and negative supercoiling, respectively) and resolve tangles (i.e., catenanes) and knots in the genetic material (Figure 1).^1,3,35−39^

### Structure and Catalytic Mechanism of Gyrase and Topoisomerase IV

Gyrase and topoisomerase IV are heterotetrameric enzymes with an A_2_B_2_ structure.^29,40^ Gyrase is composed of the GyrA and GyrB subunits.^40^ The analogous subunits in topoisomerase IV are ParC and ParE in Gram-negative species (originally named because of the partitioning defects that accompany mutations in these subunits) and GrlA and GrlB in Gram-positive species (named as “gyrase-like” proteins).^29,41^ For the sake of simplicity in this article, the GyrA/ParC/GrlA subunit will be termed the A subunit, and the GyrB/ParE/GrlB subunit will be termed the B subunit.

**Figure 2:**
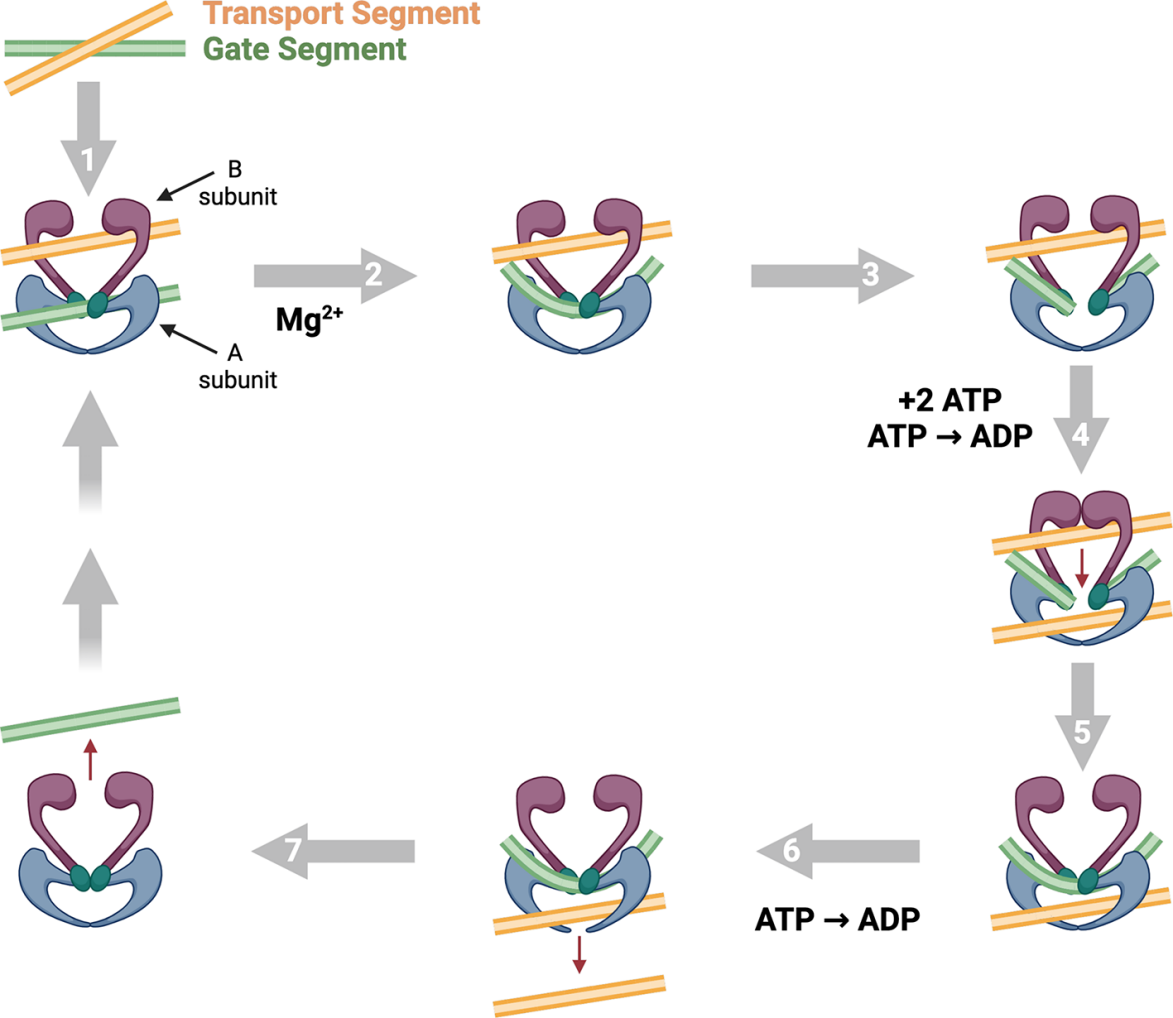
Catalytic cycle of bacterial type II topoisomerases. The double-stranded DNA passage reaction can be separated into discrete steps: 1) Capturing two segments of DNA, the gate, or G-segment (green), and the transport, or T-segment (yellow); 2) Bending the G-segment to assess DNA sites for cleavability; 3) Cleaving both strands of the G-segment; 4) Binding 2 molecules of ATP, which triggers N-gate dimerization, DNA gate opening, and T-segment strand passage through the DNA gate. The rate of the DNA passage step is increased if one of the two ATP molecules is hydrolyzed; 5) Closing the DNA gate and ligating the G-segment; 6) Hydrolyzing the second ATP molecule and releasing the T-segment through the C-gate; 7) Initiating enzyme turnover.

Gyrase and topoisomerase IV modulate the topological state of the bacterial chromosome by passing an intact double helix (the transport- or T-segment) through a transient double-stranded break that it generates in a separate segment of DNA (the gate- or G-segment).^1,3,38,42,43^ This reaction is known as the double-stranded DNA passage reaction and can be divided into a number of discrete steps (Figure 2). (1) The enzymes bind their DNA substrates.^44−49^ The first double helix bound becomes the G-segment, which interacts with both the A and B subunits. The T-segment is then captured in the N-terminal cavity of the B subunit.^48−52^ (2) The enzymes select their site of DNA cleavage by their ability to bend the G-segment.^47,53−55^ DNA segments that cannot be bent by the enzymes are not cleaved. The ability of type II enzymes to bend DNA does not reflect the intrinsic malleability of the DNA segment; rather, it is due to specific interactions between the enzyme and the G-segment.^42,47,54,55^ (3) The active site tyrosine residue on each of the two A subunits initiates a nucleophilic attack on the opposite strands of the double-helix, resulting in a covalent bond between the 4′-hydroxyl group of the tyrosine and the newly created 5′-terminal phosphate of the DNA backbone.^42,43,47,56,57^ The scissile bonds on the Watson and Crick strands are across the major groove from one another, and the cleavage reaction generates 4-base 5′-overhanging cohesive ends.^42,57^ This covalent enzyme-cleaved DNA complex is known as the cleavage complex.^1,3^ The cleavage complex plays two critical roles; it preserves the energy of the DNA sugar–phosphate backbone as well as genomic integrity while the DNA is cleaved.^38,43^ The enzymes utilize a noncanonical two metal ion mechanism to support the DNA cleavage reaction.^57−60^ As opposed to the canonical mechanism, one divalent metal ion, generally believed to be Mg^2+^, stabilizes the transition state and promotes DNA cleavage and religation, while the other anchors the DNA.^57,59,61^ The metal ions are coordinated by the TOPRIM (topoisomerase/primase) domain of the B subunit.^42^ (4) Upon ATP binding to the B subunit, the DNA gate formed by cleavage of the G-segment opens, the N-terminal portions of the two B subunits dimerize, and the T-segment is passed through the open gate.^52,61−66^ The rate of the DNA passage step is increased if one of the two ATP molecules is hydrolyzed.^67^ (5) The DNA cleavage reaction is reversed, the covalent bond between the enzyme and the DNA is broken, and the double helix is religated.^42,61^ (6) The second ATP molecule is hydrolyzed,^68^ leading to extrusion of the T-segment and (7) enzyme turnover.^69^

### Biological Functions of Gyrase and Topoisomerase IV

**Figure 3:**
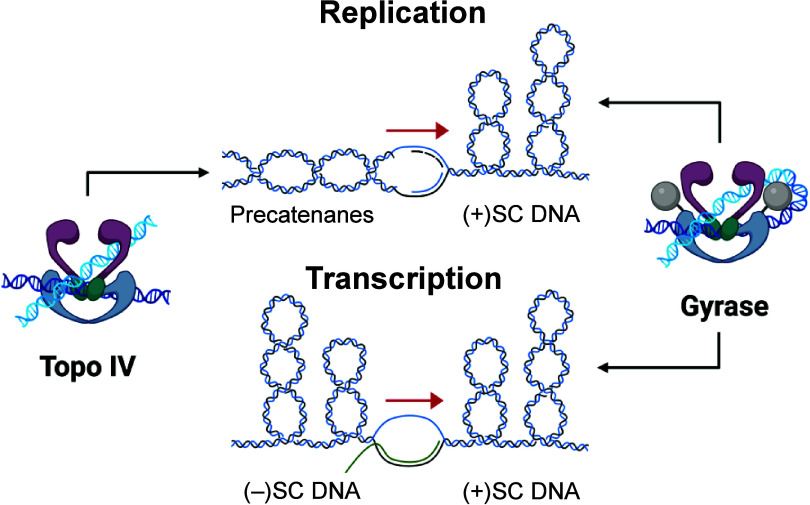
Activities of gyrase and topoisomerase IV during DNA replication and transcription. The movement of replication forks and transcription complexes along the double helix generates topological stress ahead of and behind these systems. The positive supercoils formed in front of DNA tracking machineries pose a physical barrier to progressing complexes. Gyrase (right) uses a DNA wrapping mechanism to rapidly remove these positive supercoils. The precatenanes (two intertwined partially replicated DNA duplexes) that trail replication complexes are untangled by topoisomerase IV (using a canonical strand passage mechanism) prior to cell division. The negative supercoils that accumulate behind transcription complexes are most likely removed by the ω protein, a type I topoisomerase.

Although the catalytic cycles of gyrase and topoisomerase IV are identical, the two enzymes differ in one critical aspect of their DNA interactions. Gyrase wraps its DNA substrate around the C-terminal domain of its A subunit to form a positive supercoil that is converted to a negative supercoil following strand passage (Figure 3).^70−73^ This wrapping mechanism has two major biological effects: 1) the preferred T- and G-segments come from the same DNA molecule and are in close proximity (i.e., within 100–150 base pairs of one another).^44,74−76^ As a consequence of this intramolecular DNA strand passage reaction, gyrase primarily modulates the superhelical state of the bacterial chromosome;^35,77^ 2) the handedness of DNA wrapping necessitates that gyrase acts in a unidirectional manner, removing positive supercoils and introducing negative supercoils into relaxed DNA (i.e., DNA that lacks torsional stress).^35,37,71,78^

Because of its DNA wrapping mechanism, gyrase primarily works ahead of DNA tracking systems, including replication forks and transcription complexes, to rapidly remove positive supercoils that accumulate as a result of helicases opening the double helix (Figure 3).^35,38,73,77,79^ The enzyme also works in conjunction with the omega protein (a type I topoisomerase that relaxes negative DNA supercoils), acting as a “supercoiling thermostat” to set the global level of DNA underwinding in the bacterial chromosome.^77,80−82^

In contrast to gyrase, the C-terminal domain of topoisomerase IV interacts with DNA but does not wrap the double helix (Figure 3).^35,70,83^ The lack of wrapping allows the enzyme to capture T- and G-segments from distal regions of the bacterial chromosome or even from entirely different chromosomes.^35,48^ This enzymatic characteristic has two important biological consequences: 1) topoisomerase IV can carry out strand passage reactions involving intermolecular DNA substrates (i.e., it can unlink tangled chromosomes);^29,35,36,84−86^ 2) intramolecular reactions that are performed by the enzyme are driven by the directionality of torsional stress, converting either under- or overwound DNA substrates into products with less torsional stress.^78,87−89^

Although topoisomerase IV can relax positive and negative supercoils (Figure 1), in cells, the enzyme primarily resolves precatenanes generated behind replication forks (Figure 3), unlinks daughter chromosomes during cell division, and removes knots formed during recombination events.^35,36,48,77,84−86,90^

### Cell Death Caused by Gyrase/Topoisomerase IV-Targeted Antibacterials

**Figure 4:**
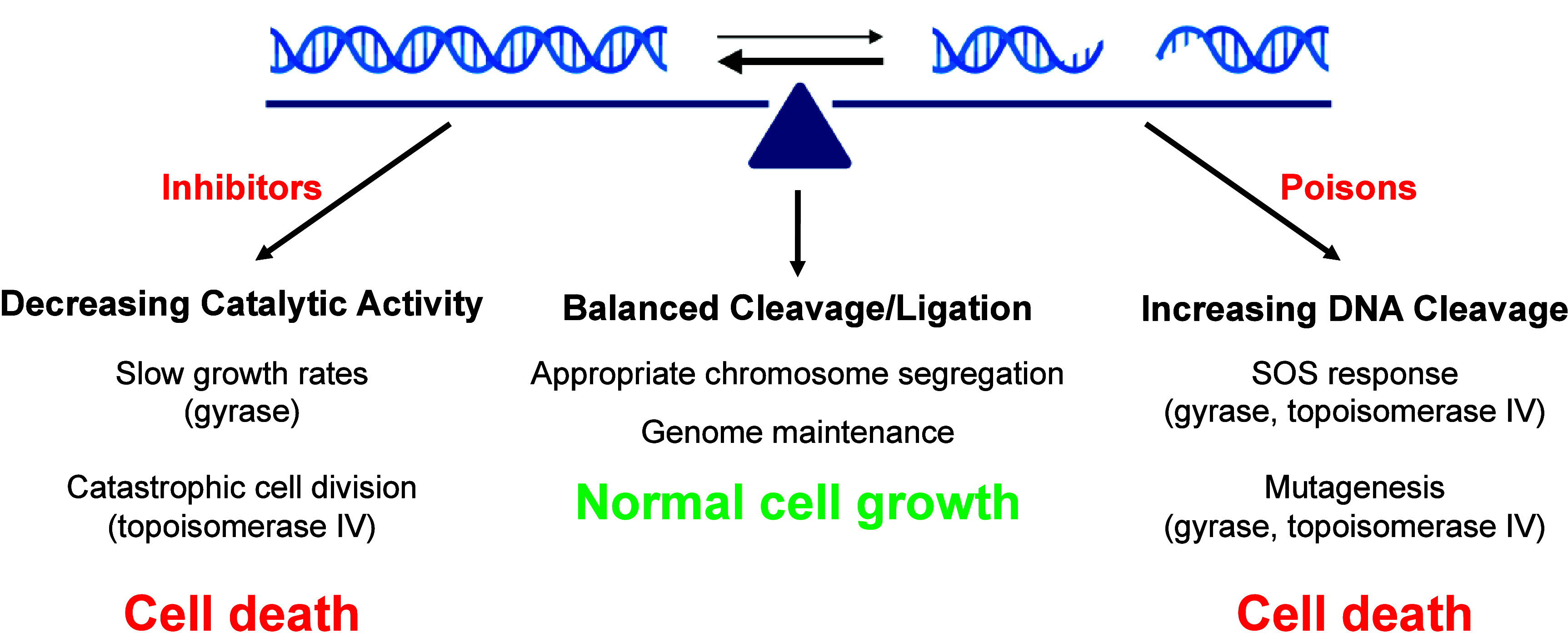
Cellular death induced by gyrase/topoisomerase IV-targeted antibacterials. Under normal cellular conditions, DNA cleavage complexes generated by gyrase and topoisomerase IV are short-lived and readily reversible, leading to normal cellular growth (middle). However, topoisomerase poisons and inhibitors shift the balance between DNA cleavage and ligation. Topoisomerase poisons increase the level of enzyme-mediated DNA breaks by stabilizing cleavage complexes. In response to DNA damage, cells initiate the SOS response. If overwhelmed by DNA damage, bacteria can undergo mutagenesis or cell death (right). Conversely, topoisomerase inhibitors prevent gyrase and topoisomerase IV from completing their catalytic cycles. This robs the cell of essential enzyme functions. Inhibition of gyrase can stall DNA replication and transcription, which impedes bacterial growth, while inhibition of topoisomerase IV can lead to catastrophic cell division and death.

Antibacterials that target gyrase and topoisomerase IV can kill cells in two different ways (Figure 4). First, because these enzymes are essential, drugs that inhibit the overall catalytic function of either gyrase or topoisomerase IV can rob the cell of critical enzymatic activities.^1,3,7,91,92^ Due to the accumulation of positive supercoils ahead of DNA tracking systems (Figure 3), inhibition of gyrase profoundly affects the ability of bacterial cells to synthesize DNA and RNA (Figure 4).^3,7,93−95^ Furthermore, inhibition of topoisomerase IV prevents the untangling of daughter chromosomes (Figure 3), leading to stalled or catastrophic cell division (Figure 4).^1,3,7,96,97^ Drugs that act by inhibiting gyrase/topoisomerase IV catalysis (without increasing levels of DNA scission) are referred to as catalytic inhibitors.^1,3,4,7,92^ Examples include coumarins, such as novobiocin, which act by inhibiting ATP binding.^7,93,98^

Second, even though gyrase and topoisomerase IV are essential enzymes, the DNA strand breaks that they generate as requisite catalytic intermediates are potentially lethal to cells.^3,7,99,100^ When the accrual of these strand breaks becomes too high, they can induce the SOS response and cell death pathways (Figure 4).^3,7,95,101−104^ Therefore, drugs that stabilize gyrase/topoisomerase IV–DNA cleavage complexes have the potential to convert these critical enzymes into potent cellular toxins that fragment the bacterial genome.^105^ Antibacterials that act by increasing levels of gyrase/topoisomerase IV-mediated DNA scission are referred to as topoisomerase poisons.^1,3,4,7,92^ Examples include fluoroquinolones, such as ciprofloxacin, which are discussed in detail in the next section.^1,3,4,7^

Three aspects of topoisomerase poisons require further discussion. First, because DNA strand breaks generated by gyrase and topoisomerase IV are covalently tethered to the enzymes, they can be ligated once the drug has dissociated from the enzyme–DNA complex.^1,3,60,106,107^ However, these stabilized cleavage complexes become more lethal when cellular machines such as replication and transcription complexes attempt to traverse the covalently attached enzymes.^3,7,99,100^ As these machines approach the cleavage complexes, they often render (by a poorly understood process) the cut DNA nonligatable by gyrase and topoisomerase IV.^3,99,106^ This results in persistent DNA breaks that must be resolved by DNA recombination processes and can lead to mutations, chromosomal abnormalities, and if unrepaired, cell death.^3,7,101−105,108^

Second, beyond the formation of DNA breaks, the presence of covalently bound enzymes on the double helix creates roadblocks that inhibit DNA tracking by replication forks and transcription complexes.^7,99,109,110^ Thus, cleavage complexes block essential genomic processes, such as replication and transcription.^3,7,99,100,109,110^

Third, because topoisomerase poisons stall the catalytic cycles of gyrase and topoisomerase IV, they also inhibit the DNA strand passage activities of these essential enzymes.^1,3,7,25^ Consequently, in addition to converting gyrase and topoisomerase IV into potentially lethal cellular toxins, topoisomerase poisons deprive bacteria of important enzymatic and genomic functions.^1,3,7,25^ There is evidence that topoisomerase poisons can kill bacteria by either the creation of gyrase/topoisomerase IV-generated DNA strand breaks or the inhibition of these important enzymes and the cellar processes that depend on their activities.^1,3,105,111^

## Fluoroquinolones

**Figure 5:**
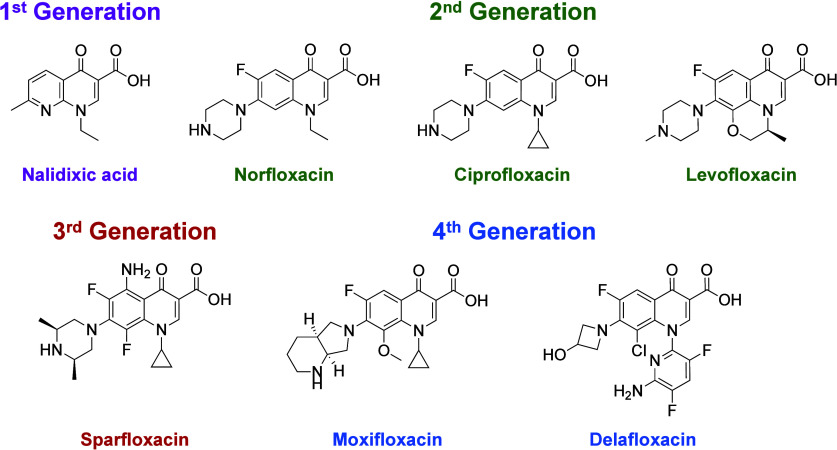
Fluoroquinolone structures. Nalidixic acid is a first-generation quinolone and the founding member of the drug class. It was approved to treat urinary tract infections but was eventually removed from the clinic due to poor pharmacodynamics. The addition of a fluorine atom at the C6 position spurred a new wave of fluoroquinolone drug development. The second-generation drugs, including norfloxacin, ciprofloxacin, and levofloxacin, had better pharmacodynamics and pharmacokinetics than their quinolone precursors and displayed activity against Gram-positive and Gram-negative pathogens. Subsequent third- (sparfloxacin) and fourth-generation (moxifloxacin and delafloxacin) drugs further improved pharmacokinetics/pharmacodynamics and extended antibacterial coverage to atypical bacteria (such as *M. tuberculosis*).

The founding member of the quinolone family, nalidixic acid, was synthesized in 1962 and introduced into the clinic for the treatment of urinary tract infections in 1964 (Figure 5).^3,112−114^ Unfortunately, due to low efficacy and poor tissue distribution, the early quinolones were dropped from clinical use.^5^ The critical change that brought quinolones back into clinical relevance was the introduction of a fluorine at the C6 position. The resulting “fluoroquinolones” displayed higher potency and superior pharmacokinetics compared with their quinolone precursors.^3,5,7^ The first clinically relevant fluoroquinolone, norfloxacin, was approved for human use in the 1980s.^115,116^ However, due to low serum levels and poor tissue distribution, its therapeutic range was limited to urinary tract infections and some sexually transmitted infections.^116,117^

The fluoroquinolone class rose to general medical prominence with the introduction of ciprofloxacin, which was the first family member to display significant activity outside of the urinary tract.^5,118,119^ Ultimately, due to its high activity against Gram-negative and some Gram-positive infections along with its improved tissue penetration, ciprofloxacin was for many years the most broad-spectrum and efficacious oral antibacterial in clinical use.^3,5,118,119^

Subsequent generations of fluoroquinolones improved pharmacokinetics and extended antibacterial coverage.^3,5,119^ Examples include levofloxacin and sparfloxacin with expanded coverage against some Gram-positive microbes, such as *Staphylococcus aureus* and *Streptococcus pneumoniae*, respectively, and moxifloxacin with substantial activity against atypical Gram-staining bacterium, such as *M. tuberculosis*.^3,5,120^ New fluoroquinolones are still being developed. For example, the newest member of this drug class, delafloxacin, was approved for clinical use in 2017.^121^ This drug is an anionic fluoroquinolone that is effective against acute skin and skin structure bacterial infections as well as community-acquired pneumonia.^121^

### Fluoroquinolone Action, Resistance, and Targeting

As mentioned earlier, fluoroquinolones are gyrase/topoisomerase IV poisons. Like other topoisomerase poisons, these drugs have two major effects on enzyme activity. First, they enhance levels of gyrase/topoisomerase IV-generated double-stranded DNA breaks by inserting between the 3′- and 5′-termini of the DNA in the cut scissile bonds.^1,3,7,9,42,107^ Bound drugs act as “molecular doorstops” that form a physical barrier to enzyme-mediated DNA ligation.^1,3,7,9,106,107^ Second, because fluoroquinolones stall the catalytic cycle at the DNA cleavage/ligation step, they inhibit the overall catalytic activity of gyrase and topoisomerase IV.^1,3,7,9,97,107^ Either of these actions can disrupt cellular functions and lead to bacterial death.^95^

Bacterial cells that evade fluoroquinolone toxicity do so by one of three resistance mechanisms: plasmid-, chromosome-, or target-mediated resistance.^1−3,7^ Plasmid-mediated resistance is caused by the uptake of plasmids that express Qnr proteins, a variant of an aminoglycoside acetyltransferase, or efflux pumps.^1,2^ Qnr proteins are DNA mimics that bind to gyrase and topoisomerase IV and decrease fluoroquinolone sensitivity by preventing the type II enzymes from binding to their DNA substrates or by blocking drug binding to the cleavage complex.^1,2,122−124^ The mutant aminoglycoside acetyltransferase, aac(6′)-Ib-cr, acetylates the unsubstituted nitrogen of ciprofloxacin, which decreases drug activity.^1,2,124−126^ Finally, plasmid-encoded efflux pumps, including QepA1 and QepA2, contribute to resistance in humans by reducing cellular levels of fluoroquinolones.^1,2,124,127,128^

Chromosome-mediated resistance is associated with reduced expression of porin diffusion channels in Gram-negative bacteria and increased expression of efflux pumps in Gram-positive bacteria.^1,2,124,129^ Either of these alterations in chromosomal genes lowers the concentrations of fluoroquinolones in bacterial cells.^1,2^

Although the above plasmid- and chromosome-mediated changes in expression patterns lead to low levels of fluoroquinolone resistance, the decreased drug sensitivity can allow further resistance mutations to accumulate.^1−3,7,130^ By far, resistance mutations in the targets for fluoroquinolones, gyrase and topoisomerase IV, are the most common and important mechanism for fluoroquinolone resistance in clinical infections.^1,3,131,132^ Therefore, the remainder of this section will focus on the targeting of this drug class to gyrase and topoisomerase IV and target-mediated fluoroquinolone resistance.

On the basis of resistance to nalidixic acid in *E. coli*, gyrase was reported to be the target of quinolone antibacterials in 1977.^133,134^ Following the identification of topoisomerase IV, cellular experiments were carried out to determine the role of this latter type II topoisomerase in quinolone targeting. To this end, *E. coli* strains were engineered that encoded resistance mutations in GyrA, the corresponding alterations in ParC, or mutations in both subunits. Compared to wild-type strains, mutations in GyrA alone conferred ∼10-fold resistance in this Gram-negative bacteria, equivalent mutations in ParC alone had no effect on fluoroquinolone potency, and simultaneous mutations in both enzymes conferred ∼50–100-fold resistance.^96^ Subsequently, it was concluded that gyrase was the primary target and topoisomerase IV was the secondary cytotoxic target of this drug class in bacteria.

The vast majority of later studies employed a genetic approach to determine the primary and secondary target of fluoroquinolones in other bacterial species.^135−139^ The topoisomerase in which the first resistance mutations occurred was declared to be the primary target, and the topoisomerase in which subsequent mutations led to higher levels of resistance was declared to be the secondary target.^96,135−140^ Surprisingly, utilizing this genetic approach, topoisomerase IV, rather than gyrase, was determined to be the primary target for fluoroquinolones in the Gram-positive bacteria *S. aureus* and *S. pneumoniae*.^136−138^ This finding shifted the fluoroquinolone-targeting paradigm from gyrase being the primary target of fluoroquinolones in all bacteria to gyrase being the primary target in Gram-negative species and topoisomerase IV being the primary target in Gram-positive species.^141^

The paradigm was further modified by subsequent genetic experiments, which reported that for almost every fluoroquinolone in almost every other species examined (irrespective of Gram stain), gyrase is the primary cellular target.^3^ This conclusion is also supported by the analysis of clinical samples, in which the vast majority of mutations are found in gyrase, and substitutions in topoisomerase IV are rarely seen in a background of wild-type gyrase.^1,142−147^ Thus, at present, the targeting of fluoroquinolones needs to be approached on a drug-by-drug and species-by-species basis.^1,3^ One aspect of the paradigm that still holds true for fluoroquinolones, is that, with rare exceptions,^148−150^ the targeting of gyrase and topoisomerase IV is not balanced (i.e., there is a primary and secondary target).^1,3^ This unbalanced targeting has profound effects on the evolution of drug resistance; a mutation in a single type II enzyme is often sufficient to cause levels of resistance that allow cells to escape fluoroquinolone treatment or potentially acquire additional mutations (in either the primary or secondary target) that lead to highly resistant strains.^2,7,151^

### Interactions with Gyrase and Topoisomerase IV through a Water–Metal Ion Bridge

The understanding of how fluoroquinolones interact with gyrase/topoisomerase IV and the mechanism of target-mediated resistance are inextricably linked. It is well established that mutations in two highly conserved, but nonessential, amino acid residues in the A subunit of gyrase/topoisomerase IV are the primary cause of target-mediated fluoroquinolone resistance.^1,3,152−154^ The amino acids most frequently associated with this resistance are a serine residue (originally identified as Ser83 in the A subunit of *E. coli* gyrase)^152,153^ and an acidic residue (originally identified as Asp87)^154^ four amino acids downstream.^1,3^ Although it had been assumed that these residues played an integral role in mediating fluoroquinolone interactions with the type II enzymes, their specific functions remained an enigma for nearly two decades.

**Figure 6:**
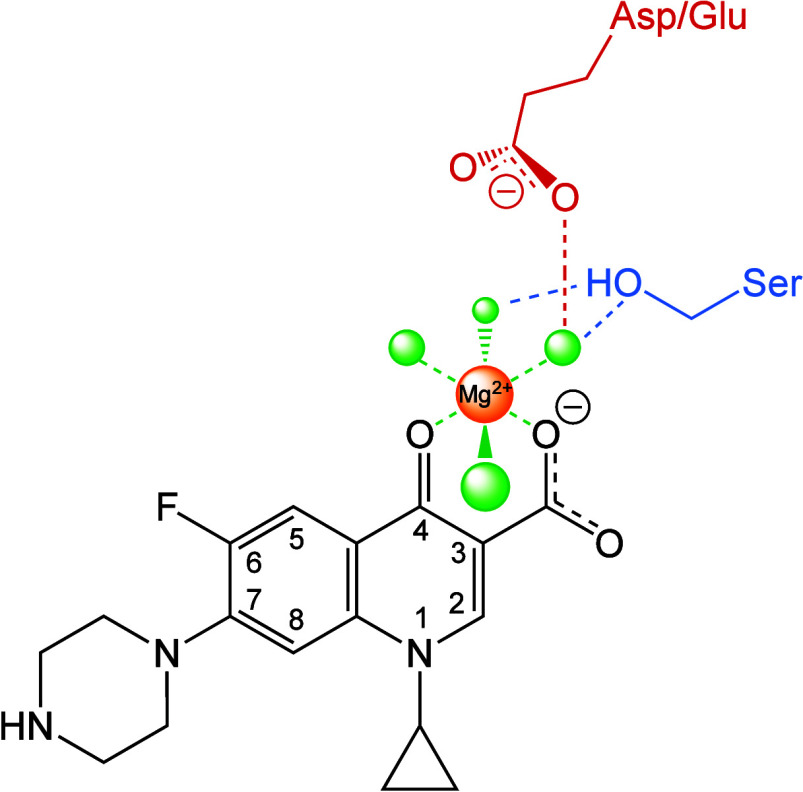
Schematic of the water–metal-ion bridge that mediates interactions between fluoroquinolones and bacterial type II topoisomerases. For simplicity, only interactions with the protein (and not the DNA) are shown. A noncatalytic divalent metal ion (orange, Mg^2+^) forms an octahedral coordination sphere (green dashed lines) between four water molecules (green) and the C3/C4 keto-acid of ciprofloxacin (black, shown as a representative fluoroquinolone). Two of the water molecules form hydrogen bonds (blue dashed lines) with the serine side chain hydroxyl group (blue), and one water molecule forms hydrogen bonds (red dashed lines) with the aspartic acid or glutamic acid side chain carboxyl group (red).

Initial structural studies of moxifloxacin/levofloxacin-induced cleavage complexes with *S. pneumoniae* topoisomerase IV and ciprofloxacin-induced cleavage complexes with *S. aureus* gyrase confirmed the insertion of fluoroquinolones in the scissile bonds of the cleaved DNA and placed the drug in the vicinity of the serine and acidic residues.^13,42,155^ However, none of the fluoroquinolones in these structures were in close enough proximity to interact directly with these amino acids. The puzzle of how mutations in the serine and acidic residues led to fluoroquinolone resistance began to be solved with the publication of a crystal structure of a moxifloxacin-induced DNA cleavage complex with *Acinetobacter baumannii* topoisomerase IV.^107^ This structure contained two fluoroquinolone molecules that were each chelated with a noncatalytic divalent metal ion. Although it had been known for many years that the C3/C4 keto acid of fluoroquinolones chelated divalent metal ions,^156^ the function of this interaction was ascribed primarily to effects on drug uptake.^157,158^ In the *A. baumannii* topoisomerase IV structure, the chelated metal ion was coordinated by four water molecules, two of which formed hydrogen bonds with the serine and acidic residues of the type II enzyme (Figure 6).^107^ This water–metal-ion interaction, which has been observed in subsequent structures,^15,159^ was proposed to bridge the fluoroquinolone directly to the residues involved in resistance.

The biological relevance of the proposed “water–metal-ion bridge” was ultimately demonstrated through a series of enzymological studies.^1,3^ These experiments showed that: 1) fluoroquinolones require a noncatalytic divalent metal ion in order to increase gyrase/topoisomerase IV-mediated DNA scission, 2) the range of divalent metal ions that can support fluoroquinolone activity is altered by mutations in the serine and acidic amino acid residues, and 3) the affinity of the chelated metal ion in the cleavage complex is diminished by mutations in these residues.^3,160−165^ These experiments also provided strong evidence that the serine and acidic residues anchor the water–metal-ion bridge as well as a direct link between fluoroquinolones and target-mediated drug resistance.^3,160−166^

The water–metal-ion bridge has been shown to play a critical role in mediating the actions of fluoroquinolones against gyrase and topoisomerase IV in every species examined to date.^1,3,160−165^ Although this bridge is the primary conduit between the drug and the type II topoisomerases, its architecture is nuanced and differs from enzyme to enzyme. In some cases, both the serine and the acidic residue are important for bridge formation,^160−162,165,167^ while in others, only one of the amino acids serves as the primary bridge anchor.^163,164^ For example, the serine residue has been replaced by an alanine residue in *M. tuberculosis* gyrase.^168,169^ As a result, the acidic residue is the major anchor for the water–metal-ion bridge.^163^ This finding explains the reduced potency of most fluoroquinolones against this enzyme.^163,168−170^

The function of the water–metal-ion bridge is dynamic and varies across type II enzymes.^1,3^ In the majority of enzymes examined, the most important role of the bridge is to serve as a vehicle for fluoroquinolone binding, as evidenced by a coordinate loss of catalytic inhibition and DNA cleavage enhancement.^160,161,163,167^ However, with some enzymes, disruption of the bridge has little effect on fluoroquinolone binding, as evidenced by sustained catalytic inhibition, but undermines the ability of the drug to enhance DNA cleavage.^162,167^ For the latter, it is assumed that the role of the bridge is to position the fluoroquinolone in the enzyme–DNA complex. In these cases, the fact that resistance tracks with the loss of DNA cleavage implies that the mechanism of drug toxicity must also be due to the induction of enzyme-generated double-stranded DNA breaks as opposed to the loss of essential enzyme activities.^1,3,7,162,167^ Thus, understanding the bridge function can provide important insights into the mechanism of fluoroquinolone cytotoxicity.

Finally, although the water–metal-ion bridge forms the primary conduit between clinically relevant fluoroquinolones and gyrase/topoisomerase IV, other ring substituents can also contribute to binding and drug specificity. For example, the inclusion of substituents at the C8 position of the fluoroquinolone backbone, such as the methoxy group in moxifloxacin, enhances the activity of these drugs against wild-type and fluoroquinolone-resistant *M. tuberculosis*.^163,171,172^

### Clinical Impact of Fluoroquinolone Resistance

Target-mediated resistance caused by mutations in residues that anchor the water–metal-ion bridge has profoundly affected fluoroquinolone use in several high-priority infectious diseases.^1,3,7,173,174^ Two examples are described below:

First, tuberculosis, which is caused by *M. tuberculosis*, is the second deadliest infectious disease in the world, surpassed only by COVID-19, in 2022.^175^ In the same year, tuberculosis caused 1.3 million deaths worldwide, and it is estimated that 1.8 billion people are infected with this pathogen globally.^175,176^ The fluoroquinolones moxifloxacin and levofloxacin have been used as second-line tuberculosis treatment for individuals who are resistant to or cannot tolerate front-line therapies,^177^ or more recently, as part of a shorter first-line regimen for multidrug-resistant tuberculosis.^178^

Because *M. tuberculosis* encodes only gyrase, it is highly susceptible to target-mediated resistance. To this point, as many as 13% of patients with tuberculosis, who are initially misdiagnosed and treated with fluoroquinolones for at least 10 days, are later found to have fluoroquinolone-resistant tuberculosis.^179^

Second, gonorrhea is a sexually transmitted infection that infects the mucosal epithelium of the genitals, rectum, and throat.^180,181^ More than 82 million new cases are observed worldwide each year.^182^ If left untreated, gonorrheal infections can cause severe complications that include pelvic inflammatory disease, infertility, and when disseminated, death.^180,183^ The etiological agent of gonorrhea is the Gram-negative bacterium, *Neisseria gonorrhoeae*.^183^

The fluoroquinolone ciprofloxacin was introduced as frontline treatment for gonorrhea in 1993.^184,185^ However, due to high levels of resistance caused by mutations in the bridge-anchoring amino acids in gyrase and topoisomerase IV, the Centers for Disease Control and Prevention (CDC) removed this drug from gonorrhea treatment guidelines in 2006.^186^ In 2021, nearly one-third of clinical *N. gonorrhoeae* isolates in the United States were resistant to ciprofloxacin, and in parts of Asia, this number exceeded 90%.^187^

### Off-Target Effects of Fluoroquinolones

Beyond the impact that target-mediated resistance has had on the use of fluoroquinolones, this antibacterial class is also associated with several off-target toxicities, most notably tendinitis, tendon rupture, and neuropathies.^188−190^ As a result, the Food and Drug Administration has issued several black box warnings regarding the use of fluoroquinolones.^191,192^ Although the underlying mechanism of these and other adverse effects is unknown, they appear to be idiosyncratic in nature and are not related to the poisoning of the human type II topoisomerases.^193^ Along with issues related to drug resistance, these safety concerns provide an even greater impetus for the discovery of new antibacterials to supplement (or eventually replace) the fluoroquinolone class.

## Novel Bacterial Topoisomerase Inhibitors, *M. tuberculosis* Gyrase Inhibitors, and Triazaacenaphthylenes

**Figure 7:**
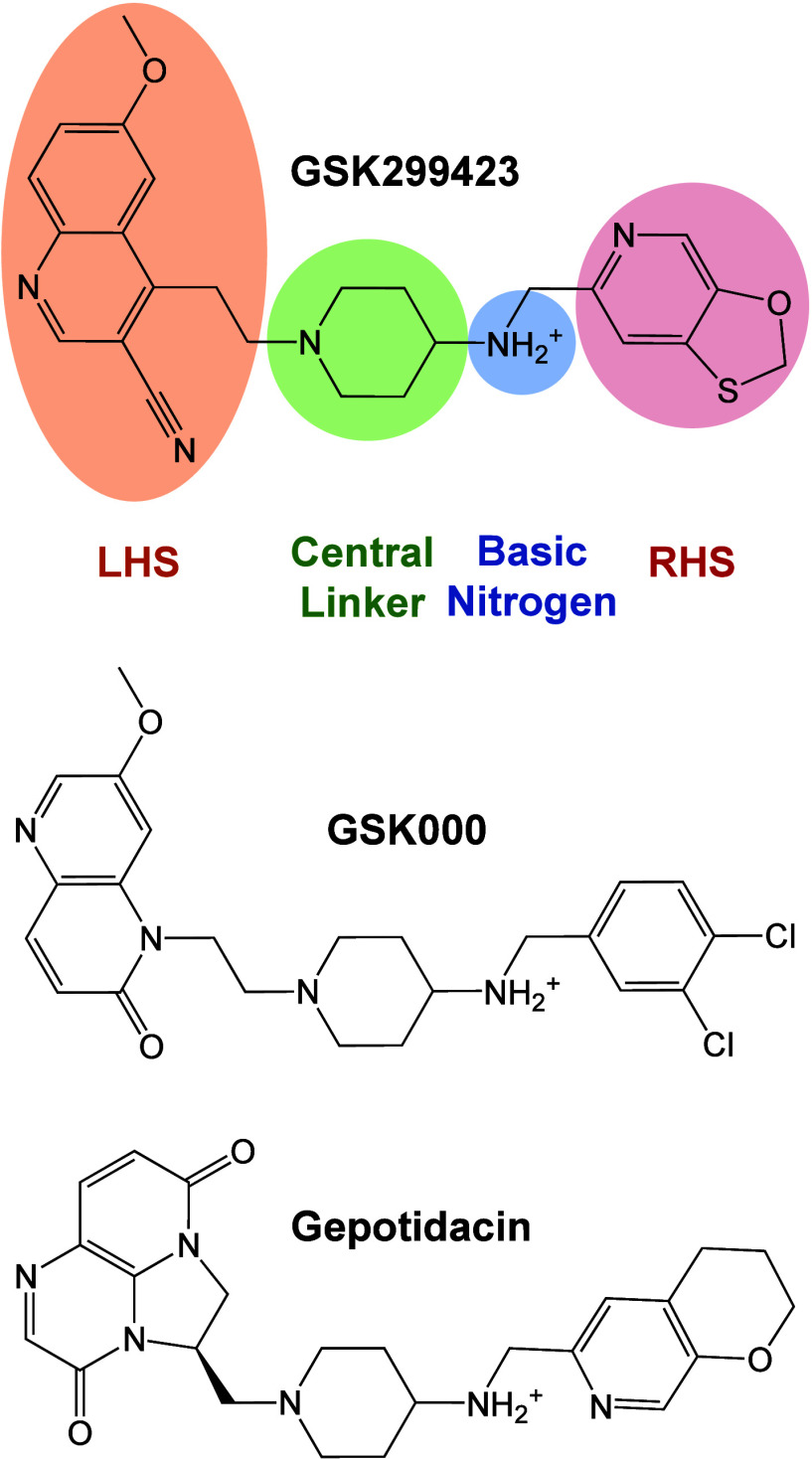
Novel bacterial topoisomerase inhibitor (NBTI), *M. tuberculosis* gyrase inhibitor (MGI), and triazaacenaphthylene structures. Members of these classes possess a left-hand substituent (LHS, orange), a central linker (green), a basic nitrogen (blue), and a right-hand substituent (RHS, red). While the LHS, central linker, and RHS are amenable to alteration, the basic nitrogen is critical for antibacterial activity. As an example, the structure of GSK299423 (top), an early piperidine-linked NBTI, is shown. GSK000 (middle) displays enhanced activity against *M. tuberculosis* gyrase and is a founding member of the MGI class. Gepotidacin (bottom) is a first-in-class triazaacenaphthylene antibacterial. Phase III trials that assessed the treatment of urinary tract infections and uncomplicated urogenital gonorrhea with gepotidacin were successfully concluded with positive outcomes.^23,24^

Novel bacterial topoisomerase inhibitors (NBTIs) were first described as a new class of bacterial type II topoisomerase inhibitors in 2007.^194,195^ The structure of NBTIs is defined by four key elements: a left-hand substituent (LHS), central linker, basic nitrogen, and right-hand substituent (RHS, Figure 7).^196,197^ Members of this class can accommodate a variety of modifications to the LHS, central linker, and RHS that still yield activity against gyrase/topoisomerase IV and bacterial cells.^196,197^ However, the basic nitrogen appears to be essential for the antitopoisomerase and antibacterial activity of this class.^13,196−200^

Although NBTIs are active against a broad range of Gram-negative and Gram-positive pathogens,^196,201−205^ the parental class generally displays poor activity against the atypical bacterium *M. tuberculosis*. Structural optimization of an NBTI that displayed moderate efficacy against tuberculosis yielded two naphthyridone/aminopiperidine derivatives with high activity against both the disease and *M. tuberculosis* gyrase.^206,207^ Because of this activity, these were designated as *M. tuberculosis* gyrase inhibitors (MGIs, Figure 7). Rather than having a broader spectrum of activity, MGIs are more specific to *M. tuberculosis* as compared to the parental class.^207^

Cardiovascular safety concerns, attributed to inhibition of the hERG potassium ion channel, proved to be a major stumbling block to the clinical development of the NBTI class.^198,208,209^ However, gepotidacin, a compound that combined minimal hERG inhibition with high antibacterial activity, emerged as the premier clinical candidate (discussed in detail below).^23,210−216^ The LHS of gepotidacin is a triazaacenaphthylene moiety; hence, this compound is considered to be the founding member of the triazaacenaphthylene class of antibacterials.^217^

Because of the mechanistic similarities between NBTIs, MGIs, and triazaacenaphthylenes, unless there is a reason to be specific, these compounds will be collectively referred to as NBTIs.

### NBTI Action, Resistance, and Targeting

In contrast to fluoroquinolones, in which two molecules bind at the active site of gyrase/topoisomerase IV (one at each scissile bond),^13,15,42,107,159^ only a single NBTI molecule binds in the gyrase–DNA complex.^13,18,72,218^ Although this is assumed to be the case for topoisomerase IV, structures with this enzyme have not yet been reported. The NBTI LHS sits in a pocket in the DNA on the 2-fold axis of the complex, midway between the two DNA cleavage sites, and the RHS sits in a pocket on the 2-fold axis between the two GyrA subunits.^13,18,72,218^ One of the most important interactions between gyrase/topoisomerase IV and the NBTI is with an aspartic acid residue (Asp82 in *E. coli* GyrA), which forms a hydrogen bond with the basic nitrogen. NBTIs do not interact directly with the serine or acidic residues that are critical for fluoroquinolone binding.^13,18,72,218^ Even though the binding sites of fluoroquinolones and NBTIs do not overlap, it appears that both drugs cannot bind to the active site of gyrase/topoisomerase IV simultaneously.^18,207,219^

As a result of their binding modality, NBTIs inhibit overall catalytic activity of gyrase and topoisomerase IV and act as topoisomerase poisons.^13,18,207,218−222^ However, in contrast to fluoroquinolones, NBTIs enhance primarily single-stranded (as opposed to double-stranded) DNA breaks.^13,18,198,207,218,220^ Furthermore, in most cases, the binding of NBTIs actually suppresses the ability of gyrase/topoisomerase IV to generate double-stranded DNA breaks.^13,18,207,218,220^ The mechanistic basis for the induction of single-stranded and suppression of double-stranded DNA breaks by NBTIs is not fully understood. However, it is believed that following cleavage of one DNA strand, the NBTI induces sufficient distortion in the active site of bacterial type II topoisomerases that it prevents these enzymes from cleaving the second strand.^13,72,207,218^

Recent results with more structurally diverse NBTIs have challenged the hallmark characteristic of this chemical class (i.e., the generation of single-stranded rather than double-stranded DNA breaks).^218,221,223,224^ Some of these compounds induce appreciable levels of double-stranded DNA breaks in addition to single-stranded breaks, although the results vary between gyrase and topoisomerase IV from different species. The basis for the enhancement of double-stranded DNA breaks by these NBTIs is an enigma, but it does not appear to be due to the binding of a second NBTI molecule in the active site of the type II enzymes.^221^

Because of the recent emergence of NBTIs as an antibacterial class with clinical potential, relatively little is known about resistance. However, mutations in gyrase/topoisomerase IV that decrease the sensitivity of laboratory strains and clinical isolates to this drug class have been reported.^198,219,225−227^ A prominent mutation occurs at the aspartic acid residue that has been shown to interact with NBTIs in structural studies.^13,18,72^ The response of NBTIs to fluoroquinolone resistance mutations varies across compounds and species. Whereas moderate to high levels of resistance are observed in some drug-species combinations, in others, such as MGIs and *M. tuberculosis* gyrase, compounds are more potent and efficacious against enzymes that carry a fluoroquinolone resistance mutation.^206,207,219,220,227,228^

As discussed above, a major drawback to fluoroquinolones is their unbalanced targeting of gyrase and topoisomerase IV, which has decreased the effectiveness of this drug class due to the development of clinical resistance.^1,2,7,151^ This drawback may be overcome by some members of the NBTI class. For example, the triazaacenaphthylene gepotidacin displays well-balanced, dual targeting against both type II enzymes in *E. coli*.^219,227,228^ Whereas there is no loss of susceptibility to gepotidacin in strains that contain a resistance mutation in either gyrase or topoisomerase IV, strains that contain a mutation in both type II topoisomerases were found to be more than 100-fold less sensitive to the triazaacenaphthylene.^219,228^ Thus, in at least some species, it appears that gepotidacin (and potentially other NBTIs) may be able to kill bacteria equally well through either gyrase or topoisomerase IV. It is assumed that this well-balanced dual-targeting of gyrase and topoisomerase IV will diminish the emergence of resistance and increase the clinical lifespan of gepotidacin and related compounds.

### Clinical Potential

Gepotidacin, a first-in-class triazaacenaphthylene, is the most clinically advanced nonfluoroquinolone gyrase/topoisomerase IV targeted-antibacterial.^23,24,210,216^ Recently, the results of phase III clinical trials for the treatment of uncomplicated urinary tract infections caused primarily by *E. coli* were reported.^23^ In these trials, gepotidacin demonstrated statistically significant superiority to an established frontline treatment for uncomplicated urinary tract infections caused by *E. coli* and other uropathogens.^23^ Thus, there is a reasonable expectation that gepotidacin will become the first new antibacterial class to treat urinary tract infections in more than two decades. This is especially significant considering that ∼50–60% of women will have a urinary tract infection in their lifetime.^229^ Beyond its potential use in treating urinary tract infections, gepotidacin was recently evaluated for a second indication. A phase III clinical trial for the treatment of uncomplicated urogenital gonorrhea with the triazaacenaphthylene was concluded in late 2023 and had positive outcomes.^24,210^ This latter study further underscores the clinical potential of gepotidacin. Finally, preclinical studies suggest that gepotidacin may have activity against a variety of biothreat pathogens, including *Bacillus anthracis*, *Francisella tularensis*, and *Yersinia pestis*, the etiological pathogens of anthrax, tularemia, and pneumonic plague, respectively.^230−233^

## Spiropyrimidinetriones

**Figure 8:**
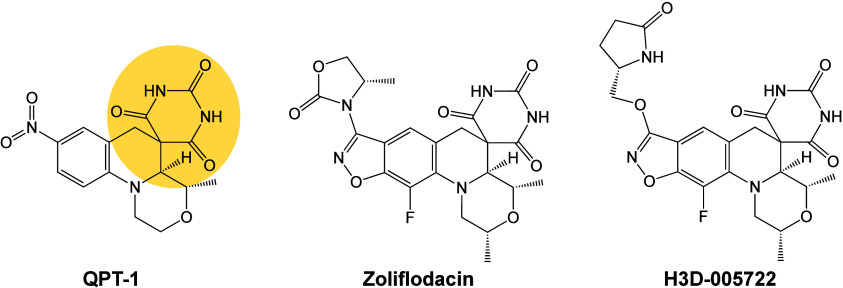
Spiropyrimidinetrione (SPT) structures. The SPT class derives its name from the spiropyrimidinetrione group (yellow) that forms critical contacts with gyrase and topoisomerase IV and is essential for antibacterial activity. Optimization of an early progenitor of this class, QPT-1 (left), led to the synthesis of zoliflodacin (middle). Phase III trials for the treatment of uncomplicated gonorrhea with zoliflodacin were successfully concluded with positive outcomes in late 2023.^20,22^ Current drug development efforts are focused on synthesizing related SPTs with high activity against *M. tuberculosis* such as H3D-005722 (right).

The most recent class of gyrase/topoisomerase IV-targeted antibacterials in clinical trials is the spiropyrimidinetriones (SPTs, Figure 8).^9,16,20,234,235^ SPTs were first identified as inhibitors of gyrase-catalyzed DNA supercoiling in 2014.^236^ Subsequently, it was determined that this class also inhibited the catalytic activity of topoisomerase IV and poisoned both type II enzymes.^14^

### SPT Action, Resistance, and Targeting

Structures of gyrase–DNA cleavage complexes formed in the presence of an SPT progenitor (QPT-1),^12^ and more recently, the clinical candidate zoliflodacin (AZD0914/ETX0914) have been published.^15,19^ The binding site for SPTs in the complex overlaps with those of fluoroquinolones. Like fluoroquinolones, SPTs insert into the cleaved scissile bonds, one molecule on each of the opposite DNA strands.^15,19^ This binding motif is consistent with the fact that SPTs induce gyrase/topoisomerase IV-mediated double-stranded DNA breaks. However, unlike fluoroquinolones, which interact with the GyrA side of the double helix, SPTs mediate their contacts with the enzyme primarily on the opposite, or GyrB side, of the double helix.^15,19,237^ Asp429 in *N. gonorrhoeae* GyrB (equivalent to Asp426 in *E. coli* GyrB), which is highly conserved across bacteria, appears to be the primary point of contact for SPTs in the enzyme–DNA complex.^15,19,237,238^ The interaction between SPTs and GyrB obviates the use of the fluoroquinolone water–metal-ion bridge.^14,15,19^

The effectiveness of SPTs (zoliflodacin and novel related compounds) against resistant bacteria and type II topoisomerases has been reported.^14,217,238−242^ Zoliflodacin has displayed potent antibacterial activity against a number of Gram-positive, Gram-negative, atypical, and anaerobic microbes, including laboratory strains and clinical isolates resistant to fluoroquinolones and other antibacterials.^217,238−241^ Furthermore, related SPTs have shown promising activity against drug-resistant strains of *M. tuberculosis* and *S. aureus* in laboratory settings.^111,243,244^ Despite the work in bacterial strains, the biochemical interactions between SPTs and fluoroquinolone-resistant type II topoisomerases have been reported only for gyrase from *N. gonorrhoeae* and *M. tuberculosis*.^14,242^ In the case of *N. gonorrhoeae* gyrase, mutations in residues that anchor the water–metal-ion bridge had no effect on the ability of the SPT to inhibit catalytic activity or enhance DNA cleavage.^14^ Moreover, both zoliflodacin and a series of novel SPT analogues either maintained or displayed higher activity against fluoroquinolone-resistant gyrase from *M. tuberculosis*.^242^ Although limited in scope, these studies provide optimism that SPTs have the potential to maintain activity against a broad spectrum of fluoroquinolone-resistant infections.

SPT resistance studies have been performed only with zoliflodacin in strains of *N. gonorrhoeae*. These studies indicate that (at least in this species) gyrase is the primary cellular target of zoliflodacin.^237^ Reported resistance mutations are located in the TOPRIM domain of the B subunit in Asp429 as well as other amino acid residues that interact (directly or indirectly) with zoliflodacin in structural studies.^19,237^ This finding supports the purported role of these residues in mediating SPT–gyrase interactions.^15,19^

Even though zoliflodacin displays high activity against purified *N. gonorrhoeae* topoisomerase IV, to date, no cellular mutations in this enzyme have been reported, even in the background of gyrase mutations.^16,237^ Although it is assumed that topoisomerase IV is a secondary target for SPTs in cells that express both type II enzymes, this has yet to be demonstrated. Nonetheless, the unbalanced cellular targeting of zoliflodacin could eventually have clinical consequences.

### Clinical Potential

Zoliflodacin is the most clinically advanced SPT. Recently, phase III trials for the treatment of uncomplicated gonorrhea with zoliflodacin were completed with positive outcomes.^20,22^ In this trial, zoliflodacin demonstrated statistical noninferiority at the urogenital site to a current global standard of care treatment.^22^ These results highlight the clinical promise of zoliflodacin to treat this prevalent sexually transmitted disease, especially existing fluoroquinolone-resistant strains. Preclinical studies with related compounds suggest that SPTs may also have potential for the treatment of tuberculosis.^9,111,242,244^

## Allosteric Gyrase/Topoisomerase IV Poisons

**Figure 9:**
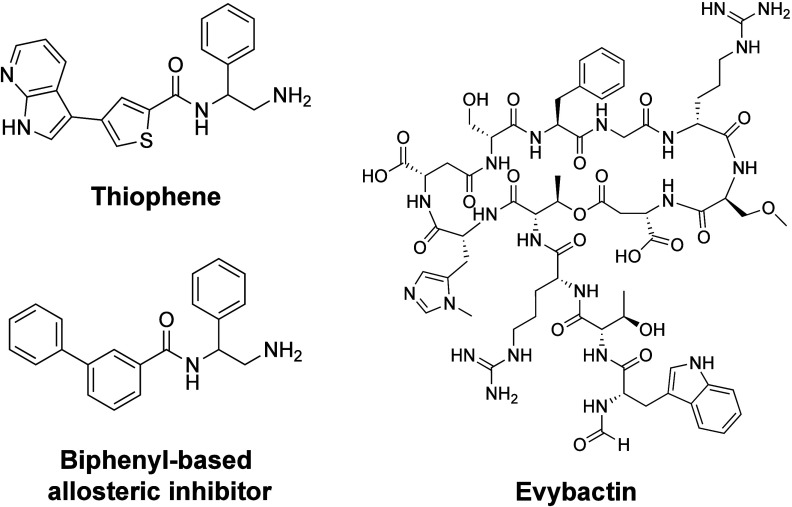
Structures of allosteric gyrase/topoisomerase inhibitors and poisons. Thiophenes (top, defined as compound 1 in Chan et al.^17^) defined a class of “allosteric” poisons that bind outside of the gyrase/topoisomerase IV active site. Recently, new allosteric inhibitors, including a biphenyl-based allosteric inhibitor (bottom, designated as compound 2 in Orritt et al.^247^) and the antibiotic evybactin (right), have been described that bind to the type II enzymes in the same pocket as members of the thiophene class.

Apart from gepotidacin and zoliflodacin, no other new gyrase/topoisomerase IV-targeted antibacterial agents have advanced to clinical trials. As discussed above, the fluoroquinolones, NBTIs, and SPTs interact with the bacterial type II topoisomerases in the DNA cleavage/ligation active site.^3,9^ Recently, a new mode of gyrase/topoisomerase IV poisoning has been described. Compounds that use this mode, which are known as “allosteric poisons,” affect enzyme catalysis and DNA cleavage through interactions outside of the active site in a binding pocket between the GyrA winged helix domain and the GyrB TOPRIM domain.^245^ Of the allosteric poisons, thiophenes and related compounds are the most well-described (Figure 9).^17,246,247^

Even though thiophenes interact distally to the cleavage/ligation active site of gyrase/topoisomerase IV, two molecules bind per enzyme heterotetramer and induce double-stranded DNA breaks.^17^ Like the NBTIs, it is presumed that the increase in DNA cleavage that accompanies thiophene binding results from distortions in the gyrase/topoisomerase IV active site. However, the specific mechanism of DNA scission enhancement is not yet known. Thiophenes are not nearly as potent as ciprofloxacin against bacterial cultures but maintain activity against strains that express fluoroquinolone-resistant gyrase/topoisomerase IV.^17^ No studies with purified fluoroquinolone-resistant enzymes have been reported.

Thiophenes have not progressed beyond preclinical development. Similar to NBTIs, the advancement of these compounds has been stymied by their inhibition of hERG cardiac potassium ion channels.^246^ Time will tell whether a new thiophene-based compound that overcomes hERG inhibition will be developed and emerge as a bona fide clinical candidate.

Recently, a series of computationally designed biphenyl-based inhibitors (Figure 9) were reported that target the same allosteric binding site on gyrase/topoisomerase IV as the thiophenes.^247^ These biphenyl compounds inhibited the catalytic activities of gyrase and topoisomerase IV and displayed antibacterial activity against laboratory strains of *E. coli* and *S. aureus*.^247^ It is not yet known whether the biphenyl-based inhibitors also poison gyrase and topoisomerase IV.

Finally, although structurally unrelated to thiophenes, the natural antibiotic evybactin (Figure 9), produced by *Photorhabdus noenieputensi*, has been shown to bind to the same allosteric pocket on gyrase/topoisomerase IV as thiophenes.^248^ Evybactin inhibits the catalytic activities of gyrase and topoisomerase IV and induces enzyme-mediated double-stranded DNA breaks in an ATP-dependent manner.^248^ However, little else is known about the interactions of this antibiotic with bacterial type II topoisomerases.

## Conclusions

Beyond their essential cellular functions, gyrase and topoisomerase IV are the targets for fluoroquinolones, which are among the most broad-spectrum and efficacious antibacterial agents used worldwide. Unfortunately, over the four decades that fluoroquinolones have been in clinical use, target-mediated drug resistance has grown to the point where it has curtailed the medical efficacy of this critically important antibacterial class against some infections. After a long hiatus, gyrase and topoisomerase IV have returned to the forefront of antibacterial development. Two new classes of compounds, triazaacenaphthylenes and spiropyrimidinetriones, have been identified that successfully treat important human infections through these validated enzyme targets. Phase III clinical trials with members of both classes, gepotidacin and zoliflodacin, were completed with positive outcomes. If approved, these compounds are poised to proceed to clinical use. Indeed, it appears that you can teach old enzymes new tricks.
